# Secondary Aroma: Influence of Wine Microorganisms in Their Aroma Profile

**DOI:** 10.3390/foods10010051

**Published:** 2020-12-27

**Authors:** Maria Carpena, Maria Fraga-Corral, Paz Otero, Raquel A. Nogueira, Paula Garcia-Oliveira, Miguel A. Prieto, Jesus Simal-Gandara

**Affiliations:** 1 Nutrition and Bromatology Group, Analytical and Food Chemistry Department, Faculty of Food Science and Technology, University of Vigo, Ourense Campus, E-32004 Ourense, Spain; maria.carpena.rodriguez@uvigo.es (M.C.); mfraga@uvigo.es (M.F.-C.); pazoterofuertes@gmail.com (P.O.); raquelnogueira36@gmail.com (R.A.N.); paula.garcia.oliveira@uvigo.es (P.G.-O.); 2 Centro de Investigação de Montanha (CIMO), Instituto Politécnico de Bragança, Campus de Santa Apolonia, 5300-253 Bragança, Portugal; 3 Department of Pharmacology, Pharmacy and Pharmaceutical Technology, Faculty of Veterinary, University of Santiago of Compostela, 27002 Lugo, Spain

**Keywords:** wine secondary aroma, fermentation, non-saccharomyces yeasts, lactic acid bacteria, volatile compounds, strain variability

## Abstract

Aroma profile is one of the main features for the acceptance of wine. Yeasts and bacteria are the responsible organisms to carry out both, alcoholic and malolactic fermentation. Alcoholic fermentation is in turn, responsible for transforming grape juice into wine and providing secondary aromas. Secondary aroma can be influenced by different factors; however, the influence of the microorganisms is one of the main agents affecting final wine aroma profile. *Saccharomyces cerevisiae* has historically been the most used yeast for winemaking process for its specific characteristics: high fermentative metabolism and kinetics, low acetic acid production, resistance to high levels of sugar, ethanol, sulfur dioxide and also, the production of pleasant aromatic compounds. Nevertheless, in the last years, the use of non-saccharomyces yeasts has been progressively growing according to their capacity to enhance aroma complexity and interact with *S. cerevisiae*, especially in mixed cultures. Hence, this review article is aimed at associating the main secondary aroma compounds present in wine with the microorganisms involved in the spontaneous and guided fermentations, as well as an approach to the strain variability of species, the genetic modifications that can occur and their relevance to wine aroma construction.

## 1. Introduction

### 1.1. Secondary Wine Aroma

The combination of two modest substrates such as grapes and microorganisms (those belonging to the grape microbiota and/or those intentionally added) results in a huge variability and diversity of wines. However, this apparently simple conjugation hinders extremely specific chemical reactions that can be modified to obtain a stunning array of aromas and flavors. The wine aroma comprises a mixture of volatiles that can account up to 800 compounds, although just few of them are odor-active [1]. This complex chemical composition can be split in terms of aromas into three different categories that are mostly related with the three production steps: grape culture, fermentation stage and transformation process, respectively [2]. 

Primary or varietal aromas, as this second name indicates, are due to the grape variety. Primary aromas belonging to the same grape variety may present different features depending on natural factors derived from weather, type of soil, fertilization, presence/absence of plagues or even the geographical location, that prompt different cultivation conditions in each area and also each year. Besides, the ripening period and the care of the collector when selecting grapes may have influence in the final primary aroma of wine [1]. Grapes are known to contain free and sugar-glycosidically-linked terpenes, being the monoterpenes and sesquiterpenes the ones that contribute with aroma and flavor. Among them, the most odoriferous monoterpenes alcohols are linalool, geraniol, nerol, citronellol, 3,6-dimethyl-1,5-octa-dien-1,7-diol, hotrienol and α-terpineol, which provide floral, fruity and citrus aroma to the wine [3,4]. Even though just few aromas have been directly associated to specific varieties, each grape variety possesses an aroma fingerprint. Monoterpene glycosides or ethers do not show significant changes in their amount during yeast fermentations. Therefore, they can be used to classify different varieties, such as Muscat and Riesling wines, by the study of their analytical composition based on just 12 monoterpene compounds [5,6,7]. However, the low concentration of these aromas (e.g., hotrienol thresholds between 18 and 400 μg/L and linalool in 50 μg/L) does not permit their sensorial appreciation at least their potential gets boosted in later steps by enzymatic reactions thus, having a major impact in the final wine aroma [8,9]. Wine has been demonstrated to have more than 800 volatile compounds with wide range of concentration, from ng/L to hundreds of mg/L [10]. In addition, other precursors that do not possess odoriferous characteristics, are involved in the development of other aroma substances (e.g., monoterpenes, diols or terpene, polyols, fatty acids, carotenoids, glycosylated precursors of aroma and volatile phenols) [1].

In the next aromatic level, yeasts and bacteria carry out the fermentation, this is the chemical reactions chain responsible for transforming grape juice into wine and providing secondary aromas to wine. Secondary aromas can be divided into pre-fermentative, those arisen due to the mechanical treatment of grapes, and fermentative, those boosted during alcoholic or malolactic fermentation processes [1,2]. The most utilized species for the alcoholic fermentation is *Saccharomyces cerevisiae*, although there are about 20 yeast genera with the same capacity such as *Saccharomycodes*, *Candida*, *Issatchenkia*, *Pichia*, *Hanseniaspora* (*Kloeckera*) or *Brettanomyces* (*Dekkera*) [3]. These non-saccharomyces species drive the aroma release by the secretion of proteins, mainly enzymes, and the synthesis of new secondary metabolites. In addition, they contribute to color wine stability and they do not use up the available sugar in must. Thus, they are strategically utilized for creating multi-starter, mixed or sequential cultures in combination with *S. cerevisiae* [8,11,12]. Normally, after the alcoholic fermentation, wine is submitted to the malolactic fermentation by the inoculation of lactic acid bacteria (LAB). During this stage, malic acid responsible for the tart taste gets decarboxylated by the action of *Oenococcus oeni* or *Lactobacillus plantarum*, two common used LAB species [8]. After this fermentation process, wine is microbiologically stabilized. Along the fermentation, the main created aromas belong to the volatile fatty acids, higher alcohols, acetate and ethyl ester categories which make evolve the aroma profile of wine [3]. These molecules are usually present at high sensory thresholds (the oxidation products of linalool possesses a perception threshold of 6000 μg/L) and their combination creates the matrix of wine aroma [3,13]. In fact, by the end of this fermentative stage, the term aroma becomes more complex from a chemical and sensorial point of view and thus, it turns into the term bouquet. Therefore, even though the final wine aroma composition is highly dependent on fermentative species and strains, the grape microbiome is gaining attention, since different works point to its relevance in the final sensorial properties of wines [8,12].

Tertiary aromas are created during the last step, aging of wine, where the storage of the final product is the main responsible for the transference of aromas and flavors to wine. The typical aging method is the use of wood barrels mostly built with different oak species such as *Quercus alba*, *Q. robur* or *Q. petraea* [14]. Wine aged in these barrels may be transferred with volatiles such as guaiacol-oak lactones or vanillin and even with furfural, 5-methylfurfural, eugenol, guaiacol, 5-hydroxymethylfurfural, 4-methylguaiacol, guaiacol and syringol, when applying different toast treatments to wood [15]. The use of different wood provides different volatiles to aged wine, for instance, brandies aged in *Quercus*-barrels were found to contain higher levels of ethyl-2-methylpropanoate, -butyrate and -octanoate and lower levels of butanoic acid, cis-β-methyl-γ-octalactone and syringol than when aged in *Castanea*-barrels [15]. In fact, apart from *Quercus*, other kind of woods such as *Acacia*, *Prunus* or *Castanea* are known to contain high concentrations of tannins, a kind of polyphenols, that are utilized to age wines since these non-volatile molecules can be transferred and may contribute to sensory properties such as color, astringency and bitterness [2,16]. 

Among the three classes of aroma, achieving an appropriate combination of secondary aromas represents the most intricate procedure. This stage implies the correct selection of yeasts and bacteria to perform the fermentation steps while avoiding wine spoiling due to cross contamination or due to the innate grape microbiota. Moreover, the high sensory threshold of the volatiles synthesized during this stage will define the wine aroma matrix. For obtaining a wine with well-defined secondary aromas and flavors, it is essential to understand how different microbial species interact with each other and which sensorial properties are capable to provide based on the metabolic pathways they develop.

### 1.2. Fermentation Implication on Wine Secondary Aroma

As aforementioned, yeasts and bacteria are responsible for the production of the secondary aroma during the pre-fermentative and fermentation processes. Naturally, in traditional winemaking, fermentation of grape juice is carried out by different yeast species following an order. The fermentation is initialized by non-saccharomyces yeasts, which is called spontaneous fermentation. However, these yeasts do not resist the increase of ethanol and so, they are commonly replaced by the strongly winery fermentative yeast *Saccharomyces cerevisiae* [17]. Nevertheless, it has been suggested that some of the non-saccharomyces species could persist from one year to another in wine and become dominant during fermentation like *S. cerevisiae* [18]. In the past, non-saccharomyces wine yeasts were considered as undesired microorganisms but in recent years it is well known that they can enhance the analytical composition and aroma profile of wine [19]. Therefore, wine fermentation can be defined as a complex process where different microorganisms coexist and microbial interactions influence the final product [20]. Non-saccharomyces yeasts can influence both the primary and secondary aroma through the production of enzymes and metabolites, respectively [19]. In this context, and for the development of the following sections, it is important to differentiate between the three types of alcoholic fermentations that can occur. Besides, malolactic fermentation is a process that some type of wines can also undergo (i.e., wines with high acidity) and consequently, causes an improvement of the aromatic profile of wines [5]. This process will be explained in the following sections.

**Figure 1:**
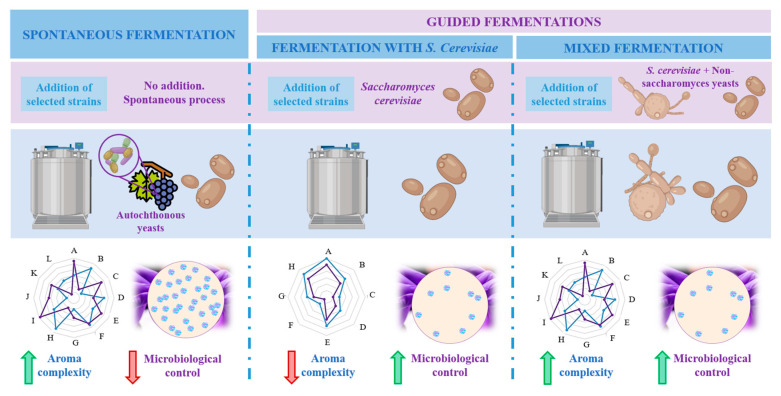
Schematic representation of the types of fermentation, the implied yeasts and the final characteristics of the obtained wine according to aroma complexity and microbiological control. Based on Reference [19].

Firstly, spontaneous fermentation is a process that naturally takes place on grape must: at the initial stages, non-saccharomyces species (already present in grapes) dominate grape juice and are then replaced by winery yeasts, commonly *S. cerevisiae*, leading to wines with a complex profile but with lower microbiological control and submitted to variability and the risk of spoilage depending on the year and the exogenous microbiota of the grapes [19]. Next, the second type of fermentation is called guided, since wine is inoculated with selected cultures named as starters which compete and limit the growth of non-saccharomyces strains [21]. This way, industrial fermentations begun to use starters of selected wine yeast strains of *S. cerevisiae* for their fermentative behaviour, their ability to enhance secondary aroma but also to achieve more uniformity in the quality of wines [19]. However, it is currently accepted that those fermentations that use more than one yeast strain can produce wine with higher quality and complexity and less content in alcohol, while microbiological control is ensured. These are called mixed fermentations [20]. Mixed cultures have shown to exert additive or synergistic effect (e.g., by metabolites exchange between yeasts) resulting in the enhancement of the chemical and sensory profile of wines [22,23]. A representation of the types of fermentations is shown in Figure 1.

It has been also highlighted that the selected inoculation strategy can modulate wine aroma profile in the case of mixed fermentations. If simultaneous inoculation is chosen, non-saccharomyces yeast and *S. cerevisiae* are added together, whereas in the case of sequential inoculation, non-saccharomyces starter is inoculated before *S. cerevisiae*, thus delaying the development of this last one [24,25]. In this sense, both strategies have shown aroma improvements depending on the utilized species. For instance, the aroma compounds resulting from the sequential fermentation of *Issatchenkia terricola* and *Pichia kudriavzevii* together with *S. cerevisiae* where higher than in the case of simultaneous fermentation [24]. In the other hand, a different experiment carried out with *Torulaspora delbrueckii* and *S. cerevisiae* showed an increase in the production of esters (fruity aroma) in the case of simultaneous fermentation when compared to sequential fermentation [26].

At last, it is worth to mention that other parameters during fermentation can also influence the wine aroma. These are temperature, molecular oxygen available during fermentation, maturation or ageing, the nitrogen source also known as yeast assimilable nitrogen (YAN) and the inoculation rate of yeasts as well as other post-fermentative parameters, such as yeasts final autolysis [27].

### 1.3. Microorganisms Implied in Wine Aroma 

**Figure 2:**
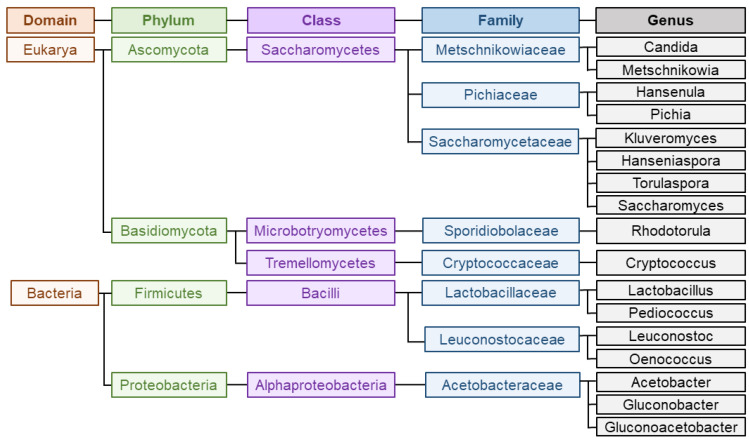
Main groups and taxonomy of the microorganisms implied in wine aroma production. Information extracted from Reference [30].

Wine is a complex matrix where the development of alcoholic fermentation, leaded by different yeasts coupled to the volatile compounds released during malolactic fermentation, leaded by LAB and acetic acid bacteria (AAB), defines wine secondary aroma [8]. Yeasts are responsible for alcoholic fermentation, and particularly, the unicellular fungi *Saccharomyces cerevisiae* governs the process, which can occur spontaneously or guided by the use of starter cultures [17]. Yeast domain counts up to more than 2000 species, among which Saccharomyces has traditionally been the most studied and important genus for industrial fermentation [8]. Within Saccharomyces species, *S. cerevisiae* is the most known since the first inoculation processes with a pure yeast culture were carried out with this species. This trend continued for many decades and resulted in the generalized use of *S. cerevisiae* as starter yeasts inmost wine fermentations [28]. However, as aforementioned, non-saccharomyces species also play an important role during fermentation. Among this group, the genera most commonly present and studied are *Hanseniaspora*, *Hansenula*, *Metschnikowia*, *Candida*, *Pichia*, *Lachancea*, *Brettanomyces*, *Kluyveromyces*, *Schizosaccharomyces*, *Torulaspora*, *Zygosaccharomyces* and *Saccharomycodes* [5,8]. In respect of bacteria, most abundant LAB belongs to genera *Lactobacillus*, *Oenococcus*, *Pediococcus* and *Leuconostoc* whereas most predominant AAB during winemaking are *Acetobacter*, *Gluconobacter* or *Gluconacetobacter* [29]. Figure 2 represents the main groups and taxonomy of the microorganisms implied in wine aroma. The challenge of winemakers and researches lies on the detection, characterization and quantification of all these microorganisms populations during fermentation to assess their participation on the development of wine secondary aroma [29].

Therefore, this review presents an overview of the main secondary aromas present in wine, the microorganisms involved in the spontaneous and guided (simultaneous or mixed) fermentations as well as an approach to the aroma variation that wine can suffer when different strains and genetic modifications have occurred. 

## 2. Compounds Involved in Secondary or Fermentative Aroma 

**Figure 3:**
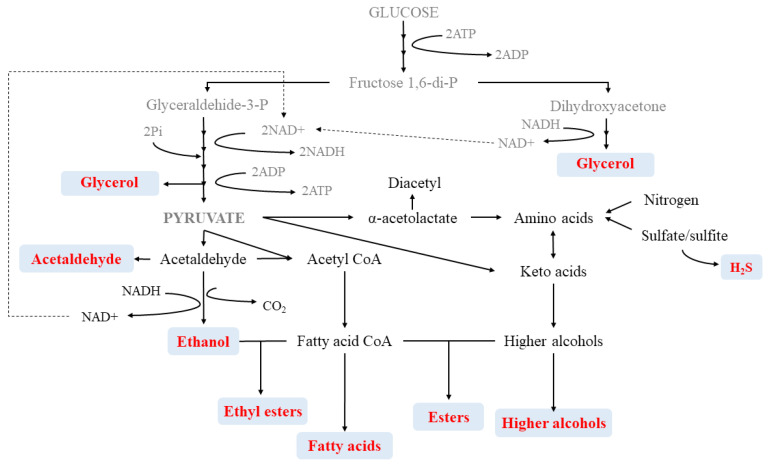
General representation of aroma compound metabolism.

The quality of wine is derived from its aroma which is, in turn, characterized by its volatile composition, mainly created during the fermentation stages. Fermentation is highly dependent on the species and strains selected but also on the components of the wine matrix. Among the main volatiles that define wine, higher alcohols, esters and fatty acids play a key role in the creation of secondary aromas (Table 1 and Figure 3).

### 2.1. Volatile Fatty Acids

In the category of aliphatic fatty acids, apart from the most abundant volatile acid, i.e., the acetic acid, the major medium chain fatty acids are hexanoic, octanoic or decanoic. Besides, in the group of the unsaturated fatty acids is worthy to mention 9-decenoic acid which possesses preservative properties and is relevant from an aroma point of view when transformed into ethyl ester [31]. 

Yeasts are the primary producers of these fatty acids which are the initial substrate for the final formation of ethyl esters. Among yeasts, *S. cerevisiae* is capable of synthesizing mainly hexanoic and octanoic acids in high amounts, but also pentanoic, decanoic and 3-methylbutanoic acids. Other non-saccharomyces species such as the genus *Hanseniaspora* has been described to produce acetic acid (in very variable ranges, from 0.6 up to 3.4 g/L) and species such as *Hanseniaspora vineae*, *H. uvarum*, *H. guilliermondii* or *Candida zemplinina* displayed higher synthesis rates for isobutyric acid [3,19]. However, it has been stated that this group of yeasts does not present a distinct biosynthesis of fatty acids. In fact, the use of mixed fermentations including *S. cerevisiae* and non-saccharomyces can modify the chemical profile of the single *S. cerevisiae* model. In general terms, this combination shows a reduction in the amount of medium-chain fatty acids, as it happens when inoculating *S. cerevisiae* with *H. osmophila*. Even though, the utilization of a mixture of *C. stellata* and *S. cerevisiae* could increase the amount of hexanoic and octanoic acids, followed *Pichia fermentas*. Similarly, the application of sequential inoculations based on *S. cerevisiae* and non-saccharomyces usually provides wines with lower concentrations of fatty acids [3]. The use of mixed or sequential fermentations can have benefits to regulate the content of these medium chain fatty acids, since their excessive presence may provide negative aromas with greasy, rancid and cheesy notes [3,32].

### 2.2. Higher Alcohols

The most abundant alcohols in wine, apart from ethanol and glycerol, are diols, higher alcohols and esters. Ethanol provides viscosity, balance taste and fix odors while higher alcohols and glycerol strongly contribute to the aroma complexity of wine and to the overall mouthfeel of wine. Higher alcohols are the result of the catabolism of amino acids by a process known as Ehrlich reaction, which affect directly or indirectly to the synthesis of aroma compounds. Higher alcohols are also involved as ester precursors which are important compounds in wine aroma [5,19]. Major higher alcohols are 1-propanol, isobutanol and isoamyl alcohol. Other important volatiles are the precursors of aromatic alcohols such as 2-phenylethanol, tyrosol or tryptophol and other higher alcohols but present in lower amounts, like 2-methylbutanol-1, 3-or methyl-1-butanol-1. Moderate concentrations of some of the volatiles considered to have high odor intensity, such as 3-methyl-1-butanol, 2-phenylethanol or isoamyl alcohol, can provide positive impact in the wine providing flower, honey and fruit aroma notes. However, the higher alcohol concentration plays a key role in the complexity of the aroma composition. Optimal alcohol values under 300 mg/L provide fruity and flowery notes, whereas alcohol values above 400 mg/L become negative by adding pungent and unpleasant aromas [2,3,8,19]. Among the different fermentation parameters that affect the final concentration of alcohol in wine, yeast strain is one of the key parameters followed by temperature, pH or oxygen, apart from grape ripeness and variety [33]. Higher alcohol synthesis has been widely studied and related to different species and/or inoculation protocols to obtain wines with an equilibrated higher alcohol composition. Different works have evaluated the efficiency of *S. cerevisiae* in terms of higher alcohol production [34,35]. Generally, no significant differences have been observed for 1-propanol while isobutanol, isoamyl alcohol, 3-methyl-1-butanol or 2-phenylethanol production seems to be strain-dependent and related to the presence of *S. cerevisiae*, both as pure or mixed cultures. In general terms, *H. uvarum*, *C. zemplinina* or *P. anomala* are considered as high alcohol producers, used both as single and mixed (with *S. cerevisiae*) fermentation agents [3,8,19]. Nevertheless, the single application of non-saccharomyces yeasts has been stated to produce lower amounts of total alcohols than *S. cerevisiae* and so, a reduction of the final amount of higher alcohols when using mixed cultures [36]. Indeed, *H. osmophila*, *H. guilliermondii* and *P. membranifaciens* were demonstrated to produce lower amounts of higher alcohols when tested against *S. cerevisiae*, even though *H. osmophila* provided high levels of 2-phenylethanol and isoamyl alcohol. Similarly, for the genus *Candida*, *C. zemplinina* has been described to synthesize 2-phenylethyl, glycerol and low amounts of ethanol and acetic acid. This combination has prompted its classification as fructophilic species, whereas *C. stellata* is classified as low producer. Another study with *H. uvarum* strains displayed variability in all produced higher alcohols except for isobutanol whose production seems to be boosted by *Hanseniaspora*. Indeed, another species, *H. guilliermondii*, also has a higher production rate of isobutanol than *S. cerevisiae*. Besides, same species synthesized very low amounts of 1-propanol [3,8,19].

### 2.3. Esters

Esters are another relevant group, also responsible for the aroma complexity of wines with more than 160 representatives already identified. From a chemical point of view, they can be classified into ethyl fatty acid esters or acetate esters. In the first category, ethyl hexanoate, ethyl octanoate, and ethyl decanoate are the most abundant ones. In these molecules, ethanol represents an important contribution to their structure. In the second class, higher alcohols are essential for the formation of these esters. The major acetate esters are isobutyl acetate, amyl acetate, hexyl acetate, ethyl acetate (fruity aroma), isoamyl acetate (banana aroma) and 2-phenylethyl acetate (2PA), which has been described to provide honey, fruity and floral aromas to the wine [2,3,7]. In white wine, the main fatty acid ethyl esters include ethyl butanoate, caproate, caprylate, caprate and laurate. As other esters, they can also provide fruity tones that become softer with the increasing number of carbons in their chemical structure of the formation of these esters depends on the selection of yeast species and other fermentation parameters such as low temperatures, are [7]. Different yeasts have been used to give complexity to wines through ester production including *S. cerevisiae* but also non-saccharomyces species such as *Candida*, *Hansenula* and *Pichia* since their differential enzymatic mechanisms allow the introduction of novel aromas in wines [3]. In general terms, esters have positive effects on the aroma of young wines, especially in those with neutral flavors. Nevertheless, as it happens in the case of higher alcohols, excessive amounts of esters may induce negative effects on the quality of wine. A high concentration of esters can hidden varietal aromas and simplify the composition of aroma of the final product or spoil wine, for instance, if ethyl acetate exceeds 150–200 mg/L [2,19]. 

### 2.4. Volatile Phenols

The positive aroma notes of this group of molecules have been mainly related to the aging process where the main volatile phenols are guaiacol, 4-methyIguaiacol, 4-ethylguaiacol, phenol, o-cresol or vanillin. The enzymes involved in these metabolic steps are mainly associated with LAB, such as β-glucosidases, proteases, esterases, citrate lyases and phenolic acid decarboxylases. In fact, many malolactic fermentations take place in oak barrels even though LAB can synthesize oak-like derived compounds from non-volatile phenols present in wine. Among the non-volatile phenols present in grapes it is common to find phenolic acids (caffeic, ferulic and *p*-coumaric) or their tartaric esters (caftaric acid, feruloyl tartaric acid, *p*-coumaroyl tartaric). LAB have the capacity to metabolize cinnamic acids, such as *p*-coumaric or ferulic, that through a decarboxylation step can be transformed into 4-vinyl guajacol and 4-vinylphenol. Thus, the use of LAB to obtain these compounds before the aging step has gained attention since it can help to modify the aroma complexity of wine. LAB can transform non-volatile phenols that contribute with unpleasant aromas such as pharmacy, smoke, forest, leather or pepper, into volatile pleasant ones, such as those related to vanillin, methyl vanilla or homovainyl alcohol. Apart from those that can be synthesized during fermentation stages due to their presence in grapes, another volatile phenols not present in grapes can be found in wines, i.e., acetovanillone [1,7,31].

## 3. Saccharomyces Cerevisiae

*Saccharomyces cerevisiae* is the most known yeast regarding the winemaking process. The historical importance of this yeast comes from far below as it was the first yeast observed by Antoine van Leeuwenhoek using a primitive microscope and it was then described as a living agent of transformation by Louis Pasteur [37]. As “agent of transformation”, *S. cerevisiae* was domesticated from the production of food and beverages such as bread and wine or beer, respectively [38,39]. Apart from its traditional application in food and alcoholic beverages, *S. cerevisiae* has been also used for fuel production, for the expression of engineered designed proteins and as genetic model organism [40]. Particularly, in wine production, *S. cerevisiae* was selected and has been used for centuries due to its specific characteristics: high fermentative metabolism, suitable fermentation kinetics, low acetic acid production, resistant character against higher concentrations of sugar, ethanol and sulfur dioxide and also, the production of pleasant aromatic compounds [38,41]. Therefore, in 1890, *S. cerevisiae* cultures started to be inoculated to wine and commercial starters were introduced into the market [39]. Since this moment, different approaches have been followed up such as guided or mixed fermentations for optimizing wine production and its organoleptic characteristics [19].

*S. cerevisiae* possesses a specific metabolism that regulates the production of volatile and aroma molecules. As it can be seen in Table 1, this yeast contributes to many of the aroma compounds classes present in wine (fatty acids, higher alcohols and esters), although varietal compounds and pre-fermentative compounds also contribute to the final wine complex aroma [2,3]. Some of these groups have been intensively studied using *S. cerevisiae* fermentations and also, different enzymes have demonstrated a key role in their formation, such as alcohol acetyltransferases (Atf1p and Atf2p), isoamyl alcohol acetyltransferase or ethanol acetyltransferase (implied in the formation of acetate esters) or the acyl-CoA:ethanol O-acyltransferase, related with the production of the ethyl esters [5]. In general terms, *S. cerevisiae* produce lower amounts of higher alcohols and poorer extracellular enzymes involved in the hydrolysis of structural components when compared to other non-saccharomyces species. However, it produced higher quantities of esters or acetaldehyde [42]. Ethanol content also influences sensory characteristics, providing fruity, flowery or acid aromas to wine, in specific concentrations [5]. On the other hand, sulfur compounds have been associated with negative or unpleasant aromas thus, poorer producers of sulfur dioxide *S. cerevisiae* strains are frequently selected. Besides, terpenoids, can be *de novo* produced by *S. cerevisiae* through the mevalonic acid pathway, constituting an alternative pathway [43].

Apart from those desirable characteristics of yeasts, there are other variables that can affect or have consequences on the aroma profile [43]. For instance, in the case of sparkling wines, a recent study showed that depending on the employed strain of *S. cerevisiae* and the period of aging, different aroma profiles were obtained. The study evaluated the production of ethyl esters (sour and apple aromas) and alcohols (herbaceous, rose, sweet aromas). It was demonstrated that flocculent yeasts produced higher amounts of these volatile compounds after 3 months whereas yeasts with higher autolytic ability produced more elevated amount of esters and alcohols after 6 months [44]. Other aspect that influences different aromatic profiles is geographical origin of indigenous yeasts. Some authors have pointed out that aroma or *terroir* includes a microbial aspect, since its sensory profile varies depending on the microorganisms implied. Particularly, the specific “signature” of some *S. cerevisiae* indigenous populations is linked to certain regions and environment conditions [45,46]. In this sense, it has been found that different genotypes (original from a specific region) are related to changes in the released compounds and thus, in the aroma profile. For instance, different genotypes from New Zealand were compared and it was found that depending on the area, some genotypes produced higher amounts of β-damascenone (apple, honey and floral aromas), higher concentrations of ethyl isobutyrate and ethyl-2-methyl butanoate (apple and sweet fruit aromas) or ethyl butanoate (peach, apple and sweet aromas) [47]. Another work reported that, independently from the substrate characteristics, the production of specific aromatic compounds is related with yeast origin, showing differences in the amounts of acetic acid, acetoin, acetaldehyde, n-butanol and 2,3-butanediols, 2-methyl-1-butanol and 3-methyl-1-butanol, among others [45]. Therefore, the current thinking is that origin, genotype and phenotype of *S. cerevisiae* strains affect quality parameters of wine and has prompted the interest on selecting autochthonous yeasts over commercial ones [46]. 

Nutrients (e.g., initial nitrogen and lipids) concentration and temperature are other parameters that can influence aroma. A recent study evaluated how specific environmental conditions affect the production of volatile compounds and found that their effects depended on the target compounds. However, authors found that the strain was determinant for the effects of environmental parameters [48]. Regarding higher alcohols, initial nitrogen content played a fundamental role exerting a negative quadratic effect for 2-phenylethanol and it positively affected propanol production. In general, temperature and lipid content were positively correlated with the synthesis of isobutanol and isoamyl alcohol. Interactive effects were also found between parameters. Therefore, it has been suggested that the disposal of nitrogen sources (e.g., amino acids) and the production of aroma compounds by *S. cerevisiae* do not follows linear relationships [48,49].

## 4. Non-Saccharomyces Species

### 4.1. Yeasts

#### 4.1.1. Major Yeasts 

##### Hanseniaspora/Kloeckera

*Hanseniaspora* is a genus of apiculate yeasts whereas the name *Kloeckera* is applied to its anamorph form. Nowadays, the *Hanseniaspora/Kloeckera* group, which is naturally present in grapes, comprises ten species: *H. valbyensis*, *H. guilliermondii*, *H. uvarum*, *H. opuntiae*, *H. thailandica*, *H. meyeri*, *H. clermontiae; H. vineae*, *H. osmophila* and *H. occidentalis* [50]. This genus is widely found in grape must and is characterized by its low fermentative power but also for its production of wine volatile compounds and its contribution to wine complexity [51]. Although several groups of volatile molecules are produced in wines during their fermentation with *Hanseniaspora* spp., this genus has been characterized as high producer of volatile fatty acids, esters, aldehydes and sulfur compounds but low producer of higher alcohols [19]. The most characteristic compounds produced by *Hanseniaspora* spp. that confer positive aroma to wines are acetate esters (isoamyl acetate, ethyl hexanoate, ethyl caprylate, phenylethyl propionate, ethyl caprate, ethyl 9-decenoeate, ethyl acetate, phenethyl acetate, beta-phenylethyl acetate, benzyl acetate and 2PA) [50,52,53,54] and aldehydes, such as acetaldehyde, benzaldehyde, 4-ethylbenzaldehyde and benzene acetaldehyde [50]. In addition, some alcohols (glycerol, 1-pentanol, phenethyl alcohol, benzyl alcohol), carboxylic acids (hexanoic acid, octanoic acid) and terpenes (limonene) are implicated in the wine flavors [50,52]. 

At industrial scale, *H uvarum*, *H. vineae* and *H. guilliermondii* are the most appropriate species to achieve an intense wine flavor and aroma complexity [55]. As previously stated, these microorganisms can naturally appear and develop spontaneous fermentation or they can be inoculated in mixed fermentations with *S. cerevisiae*. These strains contribute with positive oenological properties to wines conferring mainly floral and fruity notes such as chocolate, fig and tobacco *(H. uvarum*) [56], fruity-sweet coconut and woody or vanilla aromas (*H. vineae*) [57] and rose and honey sensory markers (*H. guilliermondii*) [53]. In mixed cultures, they can contribute with an enhancement of the production of volatile compounds. For instance, *H. guilliermondii* contributed with higher levels of 2PA to wine and *H. uvarum* could increase their isoamyl acetate content, when inoculated respectively alone. On the other side, an increase of the content of other compounds such as methionol, acetic acid-3-(methylthio) propyl ester or 4-(methylthio)-1-butanol, among others, occurred when they were inoculated with *S. cerevisiae* [58]. Further, 2PA is one of the compounds more studied in terms of aroma implications within species of *Hanseniaspora.* Different researchers have found that the mixed culture of both, *H. vineae* and *H. uvarum* with *S. cerevisiae*, provoke a synergistic effect on the production of 2PA, enhancing their floral, fruity (banana, pear, apple or citric fruits, among others) and honey aromas [23,59]. In addition, mixed cultures of *H. guilliermondii* with *S. cerevisiae*, have shown an increase of higher alcohols, acetate esters and acetaldehyde, while a reduction of ethanol, hydrogen sulphide and ethyl esters, when *S. cerevisiae* was used alone [60]. Another species of this genus, in this case, *H. opuntiae* was evaluated in mixed culture and the sensory analysis of the resulting wine, showed a higher floral and sweet aroma. This increment was related to the major production of some compounds such as phenylethanol or 3-methyl-1-bu-tanol and minor levels of decanoic and octanoic acid [61]. Apiculate yeasts have sometimes been related with the release of unpleasant flavour compounds but as previously stated, they can positively influence aroma profile in certain cases [51]. Therefore, authors are cautious when considering this genus as high intra-strain variability is found regarding their production of aroma compounds [21,51].

Likewise, other parameters can influence aroma. Regarding the time of inoculation, a recent study tried to elucidate the differences in the volatile aroma compounds when occurring a sequential or a simultaneous fermentation of *H. uvarum* and *S. cerevisiae.* Kai et al. (2018) observed that volatile phenols and acetate ester levels were higher in sequential fermentation, suggesting that this could be linked to a population ratio *H. uvarum*/*S. cerevisiae* higher than 1 [62]. Another study proved that the simultaneous inoculation of *H. uvarum* and *S. cerevisiae* caused an increase of medium-chain fatty acid ethyl ester content, improving floral, berry, tropical and temperate fruity aromas whereas sequential inoculation also improved floral and tropical fruity traits but produced an unpleasant ‘‘nail polish” odor [63]. Furthermore, must or wine composition also influences aroma profile. In this sense, low initial levels of YAN in the case of Ecolly wine were related to higher levels of ethyl esters and fatty acids whereas elevated content of YAN in the case of Cabernet Sauvignon must, motivated the expression of ATF1 gene and thus, an increment of the acetate ester production [62]. 

Table 2 describes the influence of different *Hanseniaspora* strains in the wine aroma profile. In general and from a chemistry point of view, the aroma improvements are explained by the production of higher concentrations of acetate esters like 2PA and isoamyl acetate, terpenes, medium-chain fatty acid-ethyl esters, benzenoids and decrease of higher alcohols [55].

##### Candida

The genus *Candida* is a collection of approximately 150 asporogenus yeast species from which 11% are agents of human infection since when they are ingested, they can enter to the bloodstream and cause fungaemia [75]. Besides, several studies show the significant impact of *Candida* spp. on the production of metabolites that affects the flavor and aroma of wines during fermentation on its own and together with *S. cerevisiae* [17,76]. Generally, it has been related to high production of esters, sulfur compounds and higher alcohols and low production of volatile fatty acids, aldehydes and volatile phenols [19].

Among this genus, the most known and studied Candida species could be *C. albicans*, *C. stellata* (reclassified as *C. zemplinina*) or *C. pulcherrima* (telemorphic form of *Metschnikowia pulcherrima*), among others [21,51]. All these species have been studied in different times. *C. albicans* was able to produce higher levels of farnesol and farnesene (gardenia/perfume aroma) [21]. *C. stellata* was found to intensify the apricot, honey and sauerkraut aromas when used alone in monoculture and increase the production of ethyl-acetate in sequential fermentation with *S. cerevisiae* on Chardonnay wine [77]. In addition, in a recent experiment, *C. zemplinina* was used in mixed cultures with *S. cerevisiae* and produce more aliphatic alcohols, certain aldehydes and ketones and esters (hexyl acetate, ethyl hexanoate, ethyl heptanoate, ethyl dodecanoate and ethyl butanoate) providing apple, fruit, herb, sweet or waxy aromas to wine [67].

*C. pulcherrima/M. pulcherrima* are commercial starters able to induce changes in wine’s profile, especially in terpenes, volatile thiols and esters [51]. For example, it was observed that *C. pulcherrima* in mixed cultures produced higher levels of ethyl acetate and less undesirable volatile compounds [17], being ethyl acetate strongly linked to a fruity flavor in wines at levels of 0.2 g/L [78]. The quantity of isoamyl acetate formed by *C. pulcherrima* was significantly higher than that produced by non-saccharomyces yeasts in pure cultures exhibiting a sweet, fruity and banana-like aroma at levels upper 0.001 g/L [17]. In another study, *M. pulcherrima* non-flocculant strain AWRI305 was tested in mixed culture with *S. cerevisiae*. The study showed an increment in the concentration of esters (especially, ethyl acetate and 2-methylbutyl acetate) and sulfur compounds. In this case, compared with *S. cerevisiae* alone, these wines showed lower content in brown tint and higher in red fruit aroma [79]. Likewise, another study showed that *M. pulcherrima* (sequential culture with *S. cerevisiae*) produced higher content in higher alcohols (specially 3-methyl-1-butanol and 2-methylpropanol), lower amounts of acetaldehyde, a severe decrease of butyl acetate and quite higher production of volatile phenols. These changes motivated the perception of smoky and flowery notes by tasters [68]. In addition, *M. pulcherrima* has been related to the production of low-alcohol wines, a desirable characteristic for the wine industry [80]. In this context, other authors have proposed this species and *C. zeylanoides* for this purpose, as they were poorer sugar consumers and effectively reduced ethanol content. *M. pulcherrima* has shown high production of higher alcohols (isobutanol and 2-phenylathanol), ethyl propionate, ethyl acetate and diacetyle, when compared to other species thus, being potentially suitable as inoculum [81]. Other strains like *C. molischiana* could produce terpenols and alcohols from a glycoside matrix. It has been also described the production of aldehydes by *Candida krusei* and volatile phenols and sulfur compounds by other species of *Candida* genus [82].

#### 4.1.2. Minor Yeasts 

Spontaneous grape-must fermentation can also begin with the growth of other minor species belonging to genera such as Rhodotorula or Pichia, among others. These yeasts with low fermentative capacity can confer wine flavor and aroma complexity by increasing the amounts of the volatile compounds responsible for the fruity aroma, through hydrolysis of aroma precursors caused by enzymatic activity [83]. Studies reported that glycosidases from minor yeast have also remarkable potential to improve aroma complexity and regional characteristics of wine [70]. Table 2 shows various examples of non-saccharomyces yeasts and their implication in wine aroma.

##### Rhodotorula

*Rhodotorula* spp. has been referred by some authors as high producer of esters and isoamyl acetate [19]. One of the most studied species is *Rhodotorula mucillaginosa* [21]. *R. mucillaginosa* possesses high extra-cellular glycosidase activity able to convert the glycosylated form of terpenes into aromatic compounds. For example, a general increase of terpene compounds (β-damascenone, geraniol, citronellol, linalool, β-terpineol) was observed in Aglianico and Fiano wines from Italy (Irpinian wines) [69]. The application potential of a Chinese strain of *R. mucillaginosa* to wine aroma enhancement was also reported [70]. In other case, an increase in the concentration of volatile compounds (neroloxide, alpha-terpineol, farnesol, limonene, linalool, citronellol, geraniol, geranyl acetone and nerolidol) was observed in samples treated with glycosidase extracts from *R. mucilaginosa*. Moreover, the enzyme treatments improved the content of volatile phenols, C^6^ compounds (1-hexanol) and fatty acids. Terpenic compounds and benzene compounds are positive aromatic compounds, while C^6^ compound, volatile phenols and fatty acids could release unpleasant aromas, depending on their concentration on the final wines [70]. A recent study also assessed the glycosidase activity and the main compounds related with the fermentative aroma produced by *R. mucillaginosa* which were 1-butanol, isoamyl alcohol, ethyl acetate, ethyl lactate and phenyl ethyl alcohol [71].

##### Pichia

The genus Pichia has been described as producer of esters, especially ethyl acetate and isoamyl acetate [19]. The yeast *Pichia kluyveri* is usually co-inoculated together with *S. cerevisiae* since it is unable to ferment to dryness on their own [84]. The use of *P. kluyveri* to increase the levels of terpenic compounds in sequential fermentations with *S. cerevisiae* had been previously reported [55]. This study also described that this yeast was able to produce high levels of esters, specially 2PA and ethyl octanoate [55]. The characteristic fruity, rose, sweet, honey flavors of wine and other grape-derived alcoholic beverages are primarily due to 2PA and ethyl octanoate, thus they provide pineapple, pear, soapy odors [72]. It has been also reported that *P. anomala* wine yeasts produce increased concentrations of esters with a fruity aroma. The yeast was found to be a potent isoamyl acetate producer and the characteristic banana-like aroma of wine was primarily due to this compound [53]. Other study showed that *P. kudriavzevii* in mixed cultures exhibit a chemical profile with higher levels of glycerol, ethyl acetate and isoamyl acetate and less content in fatty acids, higher alcohols and phenyl ethanol. These profile resulted in a floral, sweet and fruity aromas [61]. Similarly, a recent study showed that wines produced in simultaneous fermentation with *P. kudriavzevii* and *S. cerevisiae* had lower volatile acidity, higher amounts of esters and lower higher alcohols, fatty acids, benzene derivatives and C^6^ compounds concentration. In addition, the aroma profile and whole flavor and quality were improved and wines obtained higher scores in fruity and floral aromas, appearance and mouthfeel [24]. 

##### Torulaspora

As aforementioned, other genera and species have been also widely studied for their implication in the aroma profile of wine. This is the case of *Torulaspora delbrueckii* which has shown positive impact on wine’s aroma and increasingly importance [85]. *T. delbrueckii* (anamorph *Candida colliculosa*, synonym *Saccharomyces rosei*) [19] has shown strain variability regarding aroma profile and some of them exhibited low production of acetaldehyde and acetoin, both positive attributes. However, it produced small amounts of higher alcohols (being isoamyl alcohol and β-phenylethanol, the major compounds), acetate esters and ethyl esters of fatty acids. This way, it has been suggested that they slightly contribute to aroma complexity when compared to other non-saccharomyces organisms [85]. Nevertheless, a recent study investigated the effects of *T. delbrueckii* alone or in mixed culture with *S. cerevisiae* and their volatile compounds profile. In general, the presence of *T. delbrueckii* was associated with an increase of the fruity, sweet, pineapple, green apple, brandy, wine-like and strawberry sensory descriptors [22]. Other studies have related it to high production of isovaleric acid, ethyl propionate, 1-butanol and low production of acetic acid [81]. Also other authors confirm the low acetic acid production, while an increase in higher alcohols concentration (1-butanol) was observed [68]. Another study demonstrated that *T. delbrueckii* was related with higher concentrations of esters and differences were observed between mixed and sequential fermentation, promoting polyols synthesis (2,3-butanediol and 1,2-propanediol) and 1-butanol, 3-ethoxy-1-propanol and furaneol production, respectively [73]. At last, combinations of more than one species of non-saccharomyces yeasts have been also researched [19].

### 4.2. Bacteria

#### 4.2.1. Lactic Acid Bacteria 

Given the specific fermentation conditions, high ethanol production, presence of sulfur dioxide and low pH and nutrients concentrations, the environment turns out to be inhospitable for most bacteria genera. Nevertheless, lactic acid bacteria (LAB) and acetic acid bacteria (AAB) have managed to survive. LAB are in charge of the malolactic fermentation (MLF), also known as secondary fermentation which takes place in most of red wines and some white wines, performing the enzymatic decarboxylation of L-malic acid to L-lactic acid and carbon dioxide. This deacidification of wine reduces the sour taste that an excess of malic acid could give [5,86]. Simultaneously, as a result of LAB activity, volatile compounds are released enhancing aroma complexity with fruity or buttery notes, and reducing others such as vegetal or grass aroma. MLF also contributes to the microbiological stability of wine, decreasing the possibilities of spoilage by unwanted microbiota [5,87]. Within the LAB group, researchers have identified four main genera, the bacilli *Lactobacillus* and three cocci, *Oenococcus*, *Pediococcus* and *Leuconostoc* [87,88].

Among these genera, *Oenococcus oeni* is species most usually linked to MLF due to its resistance to fermentation conditions in red and white wines. [89]. It shows a well-adapted response to highly acidic wine conditions and better enzymatic activity than other selected starters [90]. Within this genus barely three have been isolated in must, *O. oenis*, *O. alcoholitolerans* [91] and *O. kitaharae* [92]. Several examples of the implications of *Oenococcus* and other LAB species is compiled in Table 3.

*Lactobacillus* genus is represented by approximately 30 species. Among them, *L. plantarum*, *L. brevis*, *L. buchneri*, *L. hilgardii* and *L. fructivorans* are the most found in must and wine. Also, other species such as *L. bobalius*, *L. casei*, *L. collinoides*, *L. fermentum*, *L. kunkeei*, *L. lindneri*, *L. mali*, *L. nagelii*, *L. oeni*, *L. paracasei*, *L. paraplantarum*, *L. uvarum* and *L. vini* have been also found [88,107]. Among them, *L. plantarum* is the most-liked by winemakers, due to its particular qualities: less nutrition requirements, lower inoculum concentration, tolerance to ethanol, high pH and sulfur dioxide and also, their diverse collection of enzymes able to enhance aroma profile in wines, such as glycosidases or esterases, among others [107,108,109]. For instance, the esterase activity of some LAB strains has been related to the increase of red- and black-berry fruit-like aroma and jam-fruit aroma as well [100].

In addition, research has been focused on the leverage of co-culture between *O. oeni* and *L. plantarum*, reporting more aroma complexity in mixed cultures [97]. For instance, a work evaluated the use of *O. oeni* and *Lactobacillus* strains and found that terpenes, norisoprenoids, phenols and vanillins were released in association, in general, with alcohol and dried sensory descriptors whereas oxidize notes were linked to phenyl-acetaldehyde and phenyl-acetic acid concentrations [96]. Furthermore, other studies have researched the application of LAB (i.e., *O. oeni*) together with mixed cultures (i.e., *S. cerevisiae* with *T. delbrueckii*). Results showed that volatile composition was quite different and scored better for global aroma than spontaneous MLF, which enhanced both pleasant and off-flavors [101]. Another study stated that *T. delbrueckii* together with *S. cerevisiae* (sequential fermentation) created more MLF favorable conditions, since lower levels of sulfur dioxide and medium chain fatty acids, promoting the development of *O. oeni* [110].

Regarding the aroma compounds related to LAB, diacetyl is the most important one. This compound is produced as a result of citric acid metabolism and can be further metabolized to 2,3-butanediol [5,88]. Therefore, citrate lyase enzyme plays an important role regulating the production of diacetyl. At low concentrations, it is related to yeasty, nutty and toasty aromas, whereas at high levels it produces sweet, buttery, creamy or milky aroma, sometimes linked to off-flavors. In this regard, some strains of *L. plantarum* do not present citrate lyase complex genes, and thus it is feasible to obtain wines with low diacetyl concentrations [5,109]. In addition, the perception of diacetyl is influenced by several factors such as the chemical composition of wines, the strain of LAB and the presence of sulfur dioxide, which can interact with diacetyl, decreasing wine’s volatility [103,109]. 

Apart from Oenococcus and Lactobacillus, different pediococci species including *P. damnosus*, *P. inopinatus*, *P. parvulus* and *P. pentosaceus* have been isolated from wines. Among them, *P. parvulus* and *P. damnosus* are more commonly found in must and wine, due to its undesirable effects in wine, being associated with unpleasant aromas, bitterness and ropiness [88]. For instance, a study performed in pinot noir wine with *P. inopinatus*, *P. parvulus* and *P. pentosaceus* reported floral and fruit-like aromas [111]. It has also been reported the existence of other species, such as the recombinant strain, *P. acidiltactici* BD16, which could improve the aroma of wine due to the production of phenolics derived from MLF [112].

*Leuconostoc* strains are known by dominating the initial fermentation stages, conducting the MLF alongside with *Lactobacillus* and *Oenococcus*. Nonetheless, as acids levels raise, *Leuconostoc* is overcome by more acid tolerant *Lactobacillus* [113]. Further, its enzymatic activity involved in flavor and aroma has been barely studied; it has been reported protease and also citrate lyase activity [114,115]. In addition, *L. paramesenteroides* was renamed as *Weissella paramesenteroides* [88]. *L. mesenteroides* is the current dominant *Leuconostoc* strain in grape juice and must. More recent studies have positively correlated the presence of *Leuconostoc* sp. with floral and buttery-like aromas [102].

#### 4.2.2. Acetic Acid Bacteria 

Acetic acid bacteria (AAB) belong to the family Acetobacteraceae. They are classified as aerobic strict gram-negative bacteria, well adapted to sugar and high ethanol environments and able to oxidize ethanol to acetic acid. AAB have been spotted on grapes and red wine; being notably higher in damage and rotten grapes and they are mainly classified in *Acetobacter* or *Gluconobacter* [116]. In contrast to LAB, AAB presence is less desirable in winemaking; they are considered as spoilage organisms due to the formation of acetaldehyde and acetic acid, among other spoilage compounds [117]. In general, low acetic acid concentration provides vinegar-like sourness, nutty and sherry-like aroma to wine, associated with a reduction in fruity characters, but as the concentration raises, the effect is replaced by an unpleasant smell [117]. The sensory threshold for acetic acid becoming undesirable depends on the wine type, e.g., in Canadian sweet wines is 1.0–1.5 g/L with an allowed maximum of 2.1 g/L [118], while in dry wine, the concentration must not exceed 1.0 g/L [119]. Another compound related to AAB metabolism that could affect wine quality is ethyl acetate, which can positively contribute to wine with floral or fruity-like aroma, though when it exceeds a specific threshold, it is also considered an undesirable compound. However, researchers have not reach an agreement about the establishment of that threshold [8].

*Acetobacter* are gram negative rods with an over-oxidative capacity, being able to oxidize ethanol to acetic acid to CO_2_ and water [119]. Generally, *A. aceti* and *A. pasteurianus* are the most often species isolated in wine [120,121]. Further, other related species are *A. cerevisiae*, *A. malorum*, *A. tropicalis* [121] or *A. rancens* and *A. suboxydans* found in Indian palm wine [122]. *Acetobacter* appearance is related to a bitter aroma and acid flavor, due to the excessive acetic acid concentration. Other aroma compounds as hexa- and octadecanoic acid ethyl esters, acetaldehyde, propionic and succinic acid have been correlated with *Acetobacter* metabolism [104].

*Gluconobacter* are also gram-negative rods, strictly aerobes, but unlike *Acetobacter* they are not able to oxidize acetate and lactate to carbon dioxide. *Gluconobacter* strains are frequently detected in grapes and can persist during the fermentation despite being relatively weak acetic acid resistant and less ethanol tolerant, since, they can be even inhibited by high levels of alcohol [106,123,124]. The presence of *Gluconobacter* has been positively correlated with the appearance of butanoic, lactic, citric and tartaric acids and other compounds such as benzyl alcohol, octanoic acid ethyl ester or ethyl 9-decenoate, among others [106]. However, it is worth mentioned that all these species together with *Acetobacter* species are majorly considered as spoilage microorganisms in wine, not used as starters and so, few investigation has been developed on their aroma implications beyond their acetic acid production.

## 5. Strain Dependent Variability and Genetics Influence on Aroma Profile 

Variability on aroma profile has been related to different factors such as soil, grapes, climate, type of fermentation, medium and involved microorganisms, among others [1]. Focusing on the microorganisms and their implication in secondary aroma, genetics helps to understand the origin of these changes based on strains genotypes and phenotypes. In this regard, it is essential to mentioned *S. cerevisiae*, as it is considered as the best understood genetic model organism and the first eukaryote genome completely sequenced, the best annotated and also likely to be genetically manipulated and analyzed [40]. *S. cerevisiae* has shown genetic divergence, as the phenotypes that are currently used have demonstrated different characteristics related to wine production, such as resistance to sulfites [125]. This fact is also explained in aroma terms, as it has been shown that wild strains of *S. cerevisiae* and other Saccharomyces species produced earthy and sulfurous characteristics, whereas wine domesticated strains produced fruity and floral notes [125]. In this sense, the development of “omics” technologies and the improvement of high throughput sequencing has deeply contributed to further study the microbial community of wines and thus, its implication in wine aroma [126]. These advances have been mostly directed towards different objectives: (1) mapping yeasts and bacteria genomes to identify new genetic variants that are responsible for desirable aroma characteristics, (2) using analytical techniques to isolate, identify and quantify volatile compounds involved in aroma profile and (3) modifying yeast and bacteria strains to obtain a specific character or ability. Further, progress is aimed at transcriptomics, proteomics, exometabolomics, etc. studies [126,127]. 

As previously stated, yeasts have been historically selected according to different characteristics: ethanol tolerance, low residual sugars levels, low volatile acids production, low nitrogen consumption or high growth rate. Frequently, these features come determined by multiple quantitative trait loci (QTL), i.e., regions linked to certain phenotypic traits [128,129]. For instance, a recent study, found in *S. cerevisiae* 51 potential QTLs related to the production of monoterpenes and found that three of them (UDV060, VLG19-I-1 and VLG3-A-1) on three different chromosomes were placed closely to genes connected to the production of aroma compounds [130]. In addition, other studies have investigated different genetic mechanisms that affect wine aroma: changes in transcription levels of ALD6 gene (involved in the conversion of acetaldehyde into acetic acid), haploinsufficiency effects on YFL040W related to acetic acid and glycerol and succinic acid production or the epistatic gene-gene interactions resulting in heterosis of FLX1 and MDH2, two genes associated to succinic acid production [128]. Other studies have tried to integrate different omics, thus identifying new genes related to wine aroma and flavor in different strains of *S. cerevisiae* and confirming the production of fatty acids and ethyl and acetate esters by using high performance liquid chromatography (HPLC), gas chromatography-mass spectrometry (GC-MS) and microarray techniques [131]. Besides, research has been performed on the influence of nitrogen availability and related gene expression of *S. cerevisiae*. It was found that depending on nitrogen levels, a total of 46 genes were up-or down-regulated, proposing some of them to be used as molecular markers. In this sense, potentially different strains could be used to obtain different aromas [27,132]. In the same context, other authors have investigated the effects on volatile compounds production and gene expression of *S. cerevisiae* when adding branched-chain amino acids to must. Different genes were identified and associated to yeast growth and amino acids transport; also 25 metabolites (higher alcohols, esters, fatty acids and branched-chain amino acid) were detected, among which 2,3-butanediol and ethyl lactate levels were highly increased. Therefore, it was suggested that the addition of branched chain amino acids was able to enhance aroma complexity [133]. All these techniques and studies have been developed to characterise different strains of *S. cerevisiae* that are tightly connected to differences in aroma compounds production, such in the case of a Gewürztraminer wine where a specific strain was able to produce increased amounts of 2-phenylethanol and cis-rose oxide and the most complex aroma profile [134]. 

On the other hand, transcriptional analyses have been also carried out on non-saccharomyces species mixed cultures with *S. cerevisiae.* It has been shown that culture in consortium of *S. cerevisiae* with other species can modify the genome transcriptional response of *S. cerevisiae* and differently express specific genes that encode for enzymes linked to the production of aroma compounds [60]. For instance, several studies have investigated *T. delbrueckii.* It has been demonstrated that the mixed cultures of these two species stimulates the transcription of some genes, such as those implied in the glucofermentative pathway, thus producing higher amounts of CO_2_ [135]. More recently, a transcriptome analysis of the same species revealed that the *T. delbrueckii* lower production of higher alcohols and acetate esters was explained by the absence of transcripts of key enzymes in those pathways whereas low levels of ethyl esters were related to down-regulation of fatty acids biosynthesis genes [136]. At last, a study bared that the presence of *T. delbrueckii* affect the transcriptional and phenotypic response of *S. cerevisiae* to ammonium nutrition by reducing its global effects. This way, mixed cultures produced higher concentrations of esters (i.e., acetic acid ethyl ester and lactic acid ethyl ester), providing fruity aroma to wine [137]. 

At last, it is also worth mention that according to the previously mention objective 3, other approaches have been explored for modifying yeasts to achieve a specific characteristic. In this sense, genetic modified organisms (GMOs) have been developed to fulfil those requirements but also other methodologies have been used to generate enhanced wine organisms not considered as GMO, such as clonal selection, random mutagenesis or sexual hybridization [129]. Further, research has been focused on grapes and its genetic base regarding the synthesis of aroma compounds during fermentation [138]. Therefore, the development of new “omics” technologies and related sciences is necessary for the elucidation of the transcriptional and genetic mechanisms involved in wine aroma formation. 

## 6. Future Perspectives and New Approaches

The fermentation of grape must and the production of premium quality wines is a complex biochemical process that involves the interactions of enzymes from many different microbial species, but mainly yeasts and LAB [139]. In recent years, the oenological industry has undergone an important transformation, becoming a sector with constant changes and innovations. As it is described before, non-saccharomyces yeasts can positively influence aroma [17,55,76]. This quality improvement allows the production of innovative and differentiated wines. These yeasts can be introduced into the winemaking process to obtain differentiated wines that reflect the characteristics of a specific region. In this context, the study of the use of non-saccharomyces autochthonous cultures to produce wines with particular oenological and sensory characteristics, would allow to choose suitable candidates to be included in commercial mixed cultures [82]. The presence of the non-saccharomyces species during the alcoholic fermentation might be of technological interest, but further studies on these yeasts for their biotechnological applications in winemaking are needed since the commercial assortment of non-saccharomyces cultures is still reduced [82]. It is yet possible to acquire some interesting species like *Torulaspora delbrueckii*, *Lachancea thermotolerans*, *Metschnikowia pulcherrima*, *Schizosaccharomyces pombe* and *Pichia kluyveri*. Other strains like *Starmerella bacillaris*, *Meyerozyma guilliermondii* and *Hanseniospora* spp., will probably be on market in coming years [55]. The use of non-saccharomyces yeasts can be also remarkable in regions where grape harvesting is put forward due to excessive rainfall and where the grape may contain insufficient amounts of aromatic compounds [70]. Bibliography shows big differences, depending on the non-saccharomyces strain employed due to the genomic diversity of those species and the importance of performing selective processes, such as those that were conducted for *S. cerevisiae* strains in the past [55]. In this sense, one parameter to consider is to identify yeast strains with a high level of β-glucosidase activity and to evaluate the hydrolysis characteristics of its enzyme extract.

Futures perspectives in the use of non-saccharomyces yeasts also aim to produce wines with lower alcohol content than those from pure *Saccharomyces* spp. starters [140]. Nowadays, consumers demand wine with low level of alcohol. Following this trend, winemakers search alternative methodologies to reduce the final content of ethanol in wine, especially in vineyards from warm climates where the grape over-ripening can occur giving an increase of sugar levels [140]. Inoculation of different non-saccharomyces yeast strains have been proposed for lowering alcohol levels in wine (<2%, depending on the yeast species and fermentation conditions) [55,141]. Different yeast species like *Hanseniaspora/Kloeckera* spp., *Pichia* spp. or *Candida* spp., which are predominant in the first stages of fermentation (up to 6% of alcohol content), consume sugars by respiration rather than fermentation. In this sense, non-saccharomyces species allow to reduce the initial ethanol content and would produce desirable levels of secondary metabolites, which will affect aroma profile [141]. Enzyme or osmotic filtration is another alternative strategy which can be used to reduce the content of ethanol in wine [140]. 

Nowadays, the production of efficient malolactic starter cultures has become another main challenge for oenological research [142]. There are several parameters to address when selecting LAB for possible use in a starter culture, such us their tolerance to acid conditions, high ethanol and SO_2_ concentrations, their compatibility with the selected yeast strains, adequate growth characteristics under winemaking conditions, the inability to produce biogenic amines and the lack of off-flavor or off-odor production [142]. Recent research highlights the importance of choosing specific LAB strains to obtain the desired wine, as specific flavors such as ethyl ester, volatile sulfides and glycosidic aroma compounds have been associated with specific strains. Since GMOs are not widely accepted by consumers, research is focused on identifying strains that can be used to modulate the aroma and flavor compounds of wine [88]. Finally, it is important to highlight that wine aroma is complex and contain an enormous chemical diversity. In this context, research should be also focused in developing simpler and non-targeted LC-MS methodology to study the entire volatile fraction of wine metabolome as well as other approaches such 2D GC, which has previously helped to solve the aroma on many complex matrices, including wine [8,143].

## 7. Conclusions 

Wine secondary aroma is complex and comes determined from the diversity of different chemical compounds. In the process of aroma formation, different factors play an important role. Different types of fermentation (single or mixed culture) and different strategies of inoculation (simultaneous or sequential) have shown differences on the final aroma profile. Further, other factors such as YAN, temperature, oxygen or time affect the sensory characteristics of wine. Regarding the whole chemical diversity found in wine and in particular, in volatile aroma compounds, those related with secondary or fermentative aroma are mainly higher alcohols and esters, together with volatile fatty acid and volatile phenols, and thus, they are the compounds mostly studied in research articles regarding wine aroma. In the last years, winemaking industry has undergone important transformations and despite *S. cerevisiae* is still used in production purposes for its desirable characteristics, non-saccharomyces yeasts have been highlighted as organisms that can positively influence aroma profile. According to provided data, there is a huge diversity of non-saccharomyces yeasts that can enhance or decrease the production of some aroma compounds, resulting in specific aroma attributes that are evaluated from a sensory point of view. Besides, MLF can affect wine aroma since LAB are tightly connected to the production of higher alcohols, esters and terpenes, together with norisoprenoids, phenols and vanillate derivatives in minor quantities. The main challenge still is to characterize their enzymatic activities and related genes. AAB are also revised since as spoilage microorganisms, they can negatively alter the aroma profile, although it has also been suggested that they can contribute with positive traits such as floral or fruity. Therefore, taking into account the diversity of yeasts and bacteria species and the necessities of the winemaking sector, genetics, transcriptomics and other sciences, aimed at decoding the strain dependent variability of species and its implications on wine aroma, are fundamental for the focused use of microorganisms and the achievement of wines with higher aroma complexity and pleasant characteristics that can fulfil the requirements of the consumers. 

## Figures and Tables

**Table 1:** Compounds involved in secondary aroma, classes of volatile aroma, main representative, desirable concentration, sensorial properties and producer microorganisms.

Aromatic Class	Main Compounds	Desirable Concentration	Sensorial Properties	Producer Organism	Ref.
Fatty acids	Acetic acid, pentanoic acid, hexanoic acid, octanoic acid, decanoic acid, 9-Decenoic acid, 3-methylbutanoic acid, sobutyric acid	200–700 mg/L	In excessive amount: rancid, greasy, and cheesy notes	*S. cerevisiae*, *P. fermentas*, *C. zemplinina*, *H. guilliermondii*, *H. vineae*, *H. uvarum*	[3,19,31]
Higher alcohols	1-Propanol-isobutanol, isoamyl alcohol, 2-Phenylethanol, tyrosol, tryptophol, 2-methylbutanol-1, 3-methyl-1-butanol-1	<300 mg/L	Floral, honey, and fruity notes (<300 mg/L). Pungent aroma (>400 mg/L)	*S. cerevisiae*, *C. zemplinin*, *H. uvarum*, *H. osmophila*, *H. guilliermondii*, *P. anomala*, *P. membranifaciens*	[2,3,5,8,19]
Esters	Ethyl hexanoate, ethyl octanoate, ethyl decanoate, ethyl acetate, isobutyl acetate, amyl acetate, hexyl acetate, 2PA, isoamyl acetate	150–200 mg/L	Fruity aroma, including banana or apple, honey, and floral tones	*S. cerevisiae*, *Candida*, *Hansenula*, *Pichia*	[2,3,7,19]
Phenolics	4-Vinyl guajacol, 4-Vinylphenol	-	Sweet vanillin aroma	LAB	[1,7,31]

**Table 2:** Different aroma compounds produced by yeast which confers good characteristics and pleasant aromatic properties to wine.

Yeast	Compounds	Matrix	Aroma (Odour Descriptor)	Ref.
*H. uvarum* and *C. stellata*	Benzyl alcohol	Cabernet sauvignon wine	Chocolate, fig and tobacco	[56]
*H. vineae*	Beta-phenylethyl acetate	Red wine from Uruguay (Tannat cultivar)	Intense fruity	[54,56]
*H. vineae*	P-hydroxybenzyl	Wine	Fruity, coconut, woody, vanilla	[56]
*H. guilliermondii*	Beta-phenylethyl acetate ester, 2PA	Wine	Rose, honey, fruity and flowery	[53]
*H. uvarum* and *H. guilliermondii*	2-phenylethanol	Grape must from Douro, Portugal	Fruity and flowery	[58]
*H. uvarum*	Ethyl acetate	Wine	Fruity	[64]
*H. uvaum*	Terpenes, C13-norisoprenoids, volatile phenols, terpineol and linalool oxide	Ecolly and Cabernet Sauvignon wine	Tropical fruity and floral	[62]
*H. vinae*	2PA, isoamyl acetate and esters	Chardonnay wine	Banana, pear, apple, citric fruits, guava	[65]
*H. vinae*	Phenyl ethyl acetate	Macabeo must	Fruity, floral and honey	[66]
*C. pulcherrima*	Ethyl acetate, Iso-amyl acetate	Wine	Fruity, sweet and banana-like	[17]
*C. zemplinina*	Hexyl acetate, ethyl hexanoate, ethyl heptanoate, ethyl dodecanoate and ethyl butanoate	Barbera wines	Apple, fruit, herb, sweet or waxy	[67]
*M. pulcherrima*	Phenol,2,6-dimethoxy	White wine	Smoky notes	[68]
*R. mucillaginosa*	Terpenic compounds (b-damascenone, geraniol, citronellol, linalool, b-terpineol)	Irpinian wines (Aglianico and Fiano wines)	Floral, sweet and ripened fruit	[69]
*R. mucillaginosa*	Terpenols	Chinese wine	Fruity and floral	[70]
*R. mucillaginosa*	C6 compounds (1-hexanol) and faty acids	Chinese wine	Grass and unpleasant fatty	[70]
*R. mucillaginosa*	3-hexene-1-ol, neroloxide, acetates and ethyl groups	Ecolly dry white wine	Citrus, sweet/acid fruit, berry, floral	[71]
*P. anomala*	Isoamyl acetate	Wine	Banana	[53]
*P. kluyveri*	2PA, ethyl octanoate	Sparkling wine	Fruity, rose, sweet, honey flavors and pineapple, pear, soapy	[55,72]
*T. delbrueckii*	Ethyl butyrate, ethyl acetate, ethyl hexanoate and ethyl hexanoate	Sparkling wine	Fruity, sweet, pineapple, green apple, brandy, wine-like, strawberry	[22]
*T. delbrueckii*	Ethyl propanoate, ethyl isobutanoate, ethyl dihydrocinnamate and isobutyl acetate	Sauvignon blanc and Merlot must	Fruitiness and complexity	[26]
*T. delbrueckii*	Isoamyl acetate, hexyl acetate, ethyl hexanoate and ethyl octanoate	Juice from Syrah grapes	Fresh and fruity	[73]
*T. delbrueckii*	3-sulfanylhexan-1-ol	Sauvignon Blanc grape must	Grapefruit/passion fruit	[74]

**Table 3:** Different aroma compounds produced by lactic and acetic acid bacteria strains.

Bacteria	Compounds	Matrix	Aroma	Ref.
**Lactic Acid Bacteria**				
*Lactobacillus brevis*	Methanethiol	Merlot wine	Unpleasant sulfur aroma	[93]
	3-(methylsulfanyl) propan-1-ol		Meaty aroma (<10 μM)	
*Lactobacillus plantarum*	Linalool, 2 phenyl-ethanol, 2,3-butanediol, 4-terpineol and geraniol	Fiano wine	Floral, fruity and spicy aroma	[94]
*Lactobacillus plantarum*	Terpenes, limonene and linalool	Synthetic wine	Flowery-citric aroma	[95]
	Benzyl alcohol and b-phenyl-ethyl-alcohol		Rose-like odor	
*Oenococcus oeni* and *Lactobacillus* spp.	Terpenes, norisoprenoids, phenols and vanillins	Synthetic wine	Alcohol and dried sensory descriptors. Fruity aroma	[96]
*Oenococcus oeni* and *Lactobacillus plantarum*	2 phenyl-ethanol, 2,3-butanediol, ethyl-lactate, terpenes and vanillate derivatives	Shiraz wine	Fruity, floral, earthy/nutty aromas	[97]
*Oenococcus oeni*	Ethyl esters	Wine	Fruit-like	[98]
*Oenococcus oeni*	2-phenylethanol, terpenes, lactic acid ethyl-ester and succinic acid, diethyl-ester	Riesling wine	Rose notes, fruity and floral notes	[99]
*Oenococcus oeni*	Hexanol, 3-methylbutylester, acid esters	Chardonnay wine	Green and herbaceous, banana notes and fruity aroma	
*Oenococcus oeni*	Substituted ethyl esters: i.e., (2S)-2-hydroxy-n-me-thylpentanoic acid	Merlot wine	Black-berry and jammy-fruit notes	[100]
*Oenococcus oeni*	Fruity esters and lower production of alcohols and terpenes	Black raspberry wine	Strong fruity and slight notes of solvent and herbaceous	[101]
*Leuconostoc*	Phenyl-ethyl acetate	Black glutinous rice wine	Sweet, floral aroma	[102]
*Leuconostoc*	2,3-butanediol		Buttery aroma	
LAB commercial starter	Diacetyl, ethyl acetate, ethyl lactate, mono-ethyl and diethyl succinate	Single-varietal red wines	Fruity, smoked/toasted.	[103]
**Acetic Acid Bacteria**				
*Acetobacter*	Ethyl esters	Highland barley wine	Fruity, grape-like aroma	[104]
	Acetic acid		Vinegar	
*Acetobacter aceti*	2PA, 3-methyl butanol, ethyl acetate	Pineapple wine	Floral-fruity aroma	[105]
*Gluconobacter*	Tartaric and citric acid, ethyl esters	Black glutinous rice wine	Acid and fruity aroma	[106]

## Data Availability

No new data were created or analyzed in this study. Data sharing is not applicable to this article.

## Abbreviations

LAB	Lactic Acid Bacteria
YAN	Yeast Assimilable Nitrogen
AAB	Acetic Acid Bacteria
2PA	2-Phenylethyl Acetate
MLF	Malolactic Fermentation
GC/MS	Gas Chromatography-Mass Spectrometry
QTL	Quantitative Trait Loci
HPLC	High Performance Liquid Chromatography
GMOs	Genetic Modified Organisms
